# "Flogging dead horses": evaluating when have clinical trials achieved sufficiency and stability? A case study in cardiac rehabilitation

**DOI:** 10.1186/1745-6215-12-83

**Published:** 2011-03-21

**Authors:** Louise Dent, Rod Taylor, Kate Jolly, James Raftery

**Affiliations:** 1 University of Southampton Clinical Trials Unit MP131, University of Southampton, SO16 6YD, UK; 2 PenTAG, Peninsula Medical School, Exeter, Devon, UK; 3 Department of Public Health, Epidemiology and Biostatistics, University of Birmingham, Birmingham, Warwickshire, UK; 4 NIHR Evaluation, Trials and Studies Coordinating Centre, University of Southampton, Southampton, SO16 7NS, UK

## Abstract

**Background:**

Most systematic reviews conclude that another clinical trial is needed. Measures of sufficiency and stability may indicate whether this is true.

Objectives: To show how evidence accumulated on centre-based versus home-based cardiac rehabilitation, including estimates of sufficiency and stability

**Methods:**

Systematic reviews of clinical trials of home versus centre-based cardiac rehabilitation were used to develop a cumulative meta-analysis over time. We calculated the standardised mean difference (SMD) in effect, confidence intervals and indicators of sufficiency and stability. Sufficiency refers to whether the meta-analytic database adequately demonstrates that an intervention works - is statistically superior to another. It does this by assessing the number of studies with null results that would be required to make the meta-analytic effect non-statistically significant. Stability refers to whether the direction and size of the effect is stable as new studies are added to the meta-analysis.

**Results:**

The standardised mean effect difference reduced over fourteen comparisons from a non-significant difference favouring home-based cardiac rehabilitation to a very small difference favouring hospital (SMD -0.10, 95% CI -0.32 to 0.13). This difference did not reach the sufficiency threshold (failsafe ratio 0.039 < 1) but did achieve the criteria for stability (cumulative slope 0.003 < 0.005).

**Conclusions:**

The evidence points to a relatively small effect difference which was stable but not sufficient in terms of the suggested thresholds. Sufficiency should arguably be based on substantive significance and decided by patients. Research on patient preferences should be the priority. Sufficiency and stability measures are useful tools that need to be tested in further case studies.

## Background

Any one clinical trial is seldom definitive by itself. Few innovative technologies have sufficient effect to be adopted on the basis of a single trial (or even without). The FDA normally authorises market access for a new drug or device based on two (or more) confirmatory trials. Evidence usually accumulates in systematic reviews and meta-analyses. Public decision making bodies such as National Institute for Health & Clinical Excellence (NICE) rely heavily on these methods. Most systematic reviews conclude that more clinical trials are needed.

Funders of non-commercial trials [1] need to consider both the state of existing knowledge and the contribution any proposed trial would make. Cumulative meta-analysis which shows the contribution of each trial has been used since 1981 [2]. Lau and Schmid et al in 1995 used it to show that more than 34,000 patients had been unnecessarily randomised into streptokinase trials for acute myocardial infarction[3].

Methods to aid the interpretation of cumulative meta-analysis have aimed to show when has sufficient information been obtained. Pogue and Yusuf in 1997 proposed using sequential monitoring boundaries with cumulative meta-analysis to assess when evidence is *"statistically significant and medically convincing" *implying additional research is not needed [4]. Such boundaries mitigate the multiplicity issues that arise from cumulative meta-analysis doing repeated analyses. Muellerleile et al in 2006 proposed an alternative method which involves calculating indicators of sufficiency and stability. Sufficiency refers to *"whether the meta-analytic database adequately demonstrates that a public health intervention works" *and stability *"refers to the shifts over time in the accruing evidence about whether a public health intervention works" *[5]. Muellerleile et al argued that stability (whether an effect has become stable across waves in a cumulative meta-analysis) was not covered by Pogue and Yusuf and their method is simpler due to not requiring prior specification of the optimum information size (which requires a researcher to have extensive knowledge of the observed results of the accumulated research before undertaking a cumulative meta-analysis). More recently, in 2008 Wetterslev et al developed Pogue and Yusuf's method by recommending ways of calculating the optimum information size for sequential monitoring boundaries [6].

This paper applies cumulative meta-analysis and Muellerleile's indicators of sufficiency and stability to twelve randomised clinical trials comparing centre-based to home-based cardiac rehabilitation between 1985 and 2007. Cardiac rehabilitation was the subject of two systematic reviews [7,8] as well as a large clinical trial in which several of the authors of this paper were involved [9,10]. We were interested in identifying the contribution of that trial to the meta-analysis and exploring research priorities.

### Aims

1. To apply cumulative meta-analysis to trials of centre-based versus home based cardiac rehabilitation, and

2. To explore using indicators of sufficiency and stability in assessing research priorities

## Methods

### Identification of studies and data extraction

We included trials identified in two previous systematic reviews [7,8,11], on home based versus centre based cardiac rehabilitation. Data extracted from these reviews included details of the trial design, participants, interventions, outcome measures, method of measurement of exercise capacity, results for each arm and standardised mean difference and 95% confidence interval. All data were checked against the original articles and the standardised mean difference and associated standard error re-calculated to check it was correct, with additional details provided by the authors. Country of trial and funder was extracted from the original trial articles. The Cochrane review defined home-based cardiac rehabilitation as "*a structured programme, with clear objectives for the participants, including monitoring, follow-up, visits, letters, telephone calls from staff, or at least self monitoring diaries" and*. centre-based cardiac rehabilitation as *"a supervised group based programme undertaken in a hospital or community setting such as a sports centre"*[7].

### Outcome measures

Our analysis was exercise capacity, the only outcome common to all trials identified in the systematic reviews. As trials reported exercise capacity in different ways, following the Cochrane review, we calculated the standardised mean difference in exercise capacity at follow up for home based rehabilitation compared to centre based rehabilitation using hedges adjusted g [12]. As some of the studies included were relatively small Hedges adjusted g was used [8].

### Cumulative meta-analysis

Cumulative meta-analysis involved updating the meta-analysis as each trial reported to show how the evidence evolved over time.

### Statistical-analysis

As in the Cochrane systematic review, the between-group differences in exercise capacity were pooled using a random effects model because of the significant clinical and statistical heterogeneity across trials. A sub-group analysis looked at those RCTs conducted in the UK.

Sufficiency was assessed by calculating the failsafe ratio as each new trial joined the cumulative meta-analysis [5]. The failsafe ratio is a measure of the number of studies with null results required to make the meta-analytic result non-statistically significant, versus the statistical significance (weight) of the evidence available already (see appendix 1 for further details). We used Muellerleile's suggested threshold for sufficiency, that is a Failsafe ratio exceeding 1 implied sufficient evidence that one form of rehabilitation was more effective than the other and that additional research was unlikely to change the weight of the evidence.

Stability was assessed by calculating the cumulative slope of the regression line of the cumulative meta-analysis results repeated over time [5]. Muellerleiles' suggested criteria, that is the cumulative slope estimate from the linear regression was less than 0.005, was used to decide if the meta-analysis was stable.

Publication bias was assessed using Begg funnel plots and by testing for funnel plot asymmetry using the Egger weighted regression test.

All statistical analyses were performed in Stata 10 (StataCorp, College Station, TX, USA).

## Results

### Included studies

The two systematic reviews included twelve studies identified up to January 2008 [9,10,13-23]. To the best of our knowledge no further relevant RCTs have been published (confirmed by a MEDLINE search on the 26^th ^August 2010).

Most of the trials included patients at low risk of another event following an acute myocardial infarction or revascularisation, excluding those with severe arrhythmias, ischemia, or heart failure [7]. Two studies included patients with New York Heart Association class 2 or 3 heart failure [18,21]

The trials involved a wide range of cardiac rehabilitation programmes which differed in frequency, duration and session length. The centre based programmes usually involved supervised exercise on cycles and treadmills. Home based rehabilitation typically focused on walking with support from a nurse or exercise specialist on the telephone. Seven studies compared comprehensive programmes whereas five included exercise only based programmes (table 1). A detailed description of the interventions included in each study can be found in Dalal et al 2010 Table three at this web link http://www.bmj.com/content/340/bmj.b5631/T3.expansion.html.

**Table 1:** Individual study results

Publication						Home based CR			Hospital based CR			Difference	
**Author**	**Country of trial**	**Funder**	**Year**	Type of intervention evaluated*	**Type of exercise capacity outcome and primary outcome**	**Mean**	**SD**	**n**	**Mean**	**SD**	**n**	**SMD**	**SE of SMD**

DeBusk-Brief [17]	USA	National Heart, Lung and Blood Institute, Bethesda, Maryland and PepsiCo Foundation, Purchase, New York	1985	Exercise based intervention only	METs (primary outcome not defined)	8	1.5	33	7.9	1.3	31	0.07	0.25

DeBusk-Extended [17]	USA	As above	1985	Exercise based intervention only	As above	7.9	1.5	33	8.9	1.4	30	-0.68	0.26

Sparks [20]	USA	Not stated. Authors from Saint Vincent Charity Hospital, Cleaveland State University; Saint Thomas Hospital, Nashville; and Ball State University, Muncie	1993	Exercise based intervention only	Peak VO_2 _max (primary outcome not defined)	1900	400	10	1950	150	10	-0.16	0.45

Bell [14]	UK	British Heart Foundation	1998	Comprehensive	Primary outcome exercise capacity (METs).	7.29	2.8	91	7.1	3.1	91	0.06	0.15

Carlson [22]	USA	Blodgett Memorial Medical Center Research Fund, East Grand Rapids, Michagan; the Kent Country Health Department Cardiovascular Mini-Grant and the Merck Pharmaceutical Educational Grant, Grand Rapids, Michagan	2000	Comprehensive, Home CR included short initial period of centre based intervention	Primary outcome peak functional capacity (METs),	7.4	1.5	34	6.8	1.7	29	0.37	0.26

Kassaian [18]	Iran	Not stated. Authors from Cardiovascular Medical Center, Vali-Asr-Ave, Tehran, Iran	2000	Exercise based intervention only	Functional capacity (METs) (primary outcome not defined)	8.9	2.9	60	12.4	2.7	65	-1.24	0.20

Arthur [13]	Canada	Heart and Stroke Foundation of Ontario	2002	Exercise based intervention only	Primary outcome exercise capacity (METs)	5.22	2.1	113	5.21	2	109	0.00	0.13

Gordon - Community [23]	USA	American Heart Association Patient Care and Outcomes Research Programme Grant, Dallas, Texas	2002	Comprehensive, Home CR included short initial period of centre based intervention	Maximal oxygen uptake (primary outcome not defined)	1.6	2.2	40	1.6	2.1	22	0.00	0.27

Gordon - Supervised [23]	USA	As above	2002	As above	As above	0.9	1.9	49	1.6	2.1	22	-0.35	0.26

Marcionni [19]	Italy	National Research Council, the University of Florence, and the Regional Government of Tuscany, Italy	2003	Comprehensive	Primary outcome total work capacity	3650.7	3957.2	74	3509.3	3343.8	79	0.04	0.09

Daskapan [16]	Turkey	Department of Physical Therapy in Ankara University, Faculty of Medicine, Turkey	2005	Comprehensive, Home CR included short initial period of centre based intervention	Exercise capacity (ml/kg/min) (primary outcome not defined)	23.6	7.4	11	23.3	6.8	11	0.04	0.43

Dalal [15]	UK	NHS Executive South West (Research and Development)	2006	Comprehensive	Secondary outcome exercise capacity (METs) Primary outcome quality of life (MacNew questionnaire)	9.7	3.1	60	7.7	2.8	44	0.66	0.20

Wu [21]	China	Not stated. Authors from Department of Physical Medicine and Rehabilitation, Taichung Veterans General Hospital, Taichung, Taiwan	2006	Exercise based intervention only	Exercise capacity (METs) (primary outcome not defined)	22.9	3.6	18	24.2	4.4	18	-0.32	0.34

Jolly (BRUM) [9,10]	UK	UK Department of Health, through the HTA programme	2007	Comprehensive	Primary outcome incremental shuttle walking test	391.3	162.1	191	407.4	157.6	179	-0.10	0.10

*Comprehensive = interventions evaluated included exercise plus education or psychological management or both. A detailed description of the hospital based and home based intervention used by each study can be found in Dalal 2010 at this link http://www.bmj.com/content/340/bmj.b5631/T3.expansion.html.  SD = Standard deviation, CR = Cardiac Rehabilitation, SMD = standardised mean difference (hedges g), SE = Standard error, METs = metabolic equivalent, VO2 max = maximum volume of oxygen.

**Figure 1:**
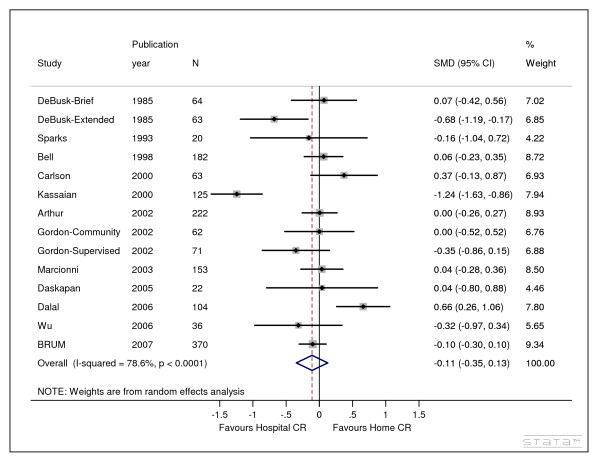
**Meta-analysis of studies published up to January 2008 comparing centre based versus home based cardiac rehabilitation - random effects model**. 1. SMD = standardised mean difference (hedges g)

The twelve studies included 14 comparisons involving 1,557 patients (Table 1). The individual study results (Figure 1) varied with six favouring centre-based cardiac rehabilitation, six favouring home and 2 favouring neither. The pooled standardised mean difference in exercise capacity was not statistically significant (random effects: SMD -0.11, 95% CI -0.35 to 0.13) (figure 1). There was evidence of high levels of statistical heterogeneity between the study results across trials. The funnel plot and associated egger regression test did not indicate evidence of small study publication bias (p-value = 0.77).

### Evolution of evidence - cumulative meta-analysis

**Figure 2:**
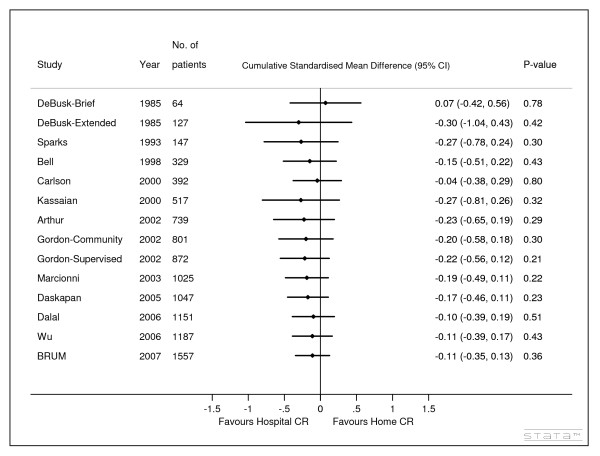
**Cumulative meta-analysis of studies published up to Jan 2008 comparing centre based versus home based cardiac rehabilitation - random effects model**. 1. The number of patients in this figure equals the cumulative number of patients included in each meta-analysis

The cumulative meta-analysis of the 14 comparisons showed the effect size and confidence interval narrowing over time, with the effect size initially favouring centre-based cardiac rehabilitation and reducing over time towards the line of no difference (figure 2). All trials except Kassaian [18] contributed to the narrowing of the confidence interval. The trend over time highlights Kassaian as a potential outlier a point also highlighted by the authors of the Cochrane review. They stated there was uncertainty due to lack of detailed reporting as to whether Kassaian compared hospital based rehabilitation to usual care instead of home based rehabilitation.

Despite BRUM being the largest and latest trial its contribution to the meta-analysis was limited to reducing the width of the confidence interval without changing the point estimate (the pooled SMD in exercise capacity prior to BRUM being published in 2007 was -0.11 (95% CI -0.39 to 0.17), post BRUM was -0.11 (95% CI -0.35 to 0.13)). The difference of -0.11 is equivalent to approximately -0.34 of a MET.

### Sufficiency and Stability

**Figure 3:**
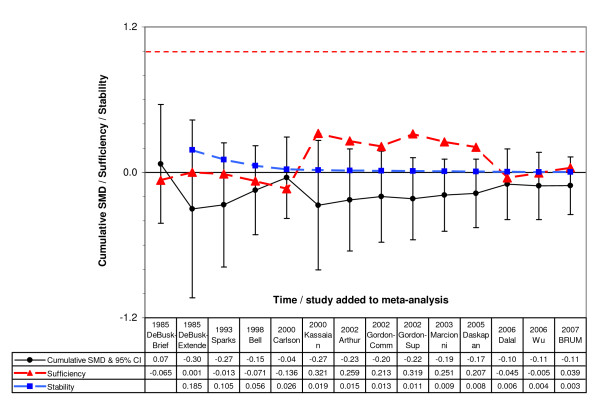
**Cumulative meta-analysis from figure 2 with indicators of sufficiency (failsafe ratio) and stability (cumulative slope)**. 1. SMD = standardised mean difference (hedges g). 2. The cumulative SMD and 95% CI shown in this figure are exactly the same as those shown in figure 2. 3. Threshold for sufficiency > 1 (shown by red dashed line), not achieved in this cumulative meta-analysis. 4. Criteria for stability < 0.005, achieved in this cumulative meta-analysis after inclusion of Wu

Figure 3 rotates figure 2 so that the slope of the mean effect indicates stability. Stability (around a null effect) was established after the results from Wu were published in 2006, 1 year before BRUM (stability 0.004 from figure 3 in 2006). This conclusion is consistent with how the point estimate in the cumulative meta-analysis changed with the addition of new studies, BRUM was the first study where the point estimate didn't change (was stable at -0.11, before and after inclusion of BRUM). Stability means that further studies are unlikely to change the aggregate picture, in this case, of a small difference.

The sufficiency indicator in figure 3 highlights two key trials: Kassaian and Dalal. Dalal's significant result favouring home based rehabilitation compensated for Kassaian's significant result favouring centre-based. The weight of evidence against the null hypothesis was strongest after the publication of Kassaian, although not sufficient (failsafe ratio = 0.321 < 1) or statistically significant and reduced greatly after inclusion of Dalal (failsafe ratio = -0.045). Sufficiency in figure 3 did not achieve Muellerleile's threshold (failsafe ratio < 1 throughout), which is unsurprising given the lack of statistical significance.

### Sensitivity analysis excluding Kassaian

Because of the uncertainty as to whether Kassaian included usual care or home based rehabilitation we conducted all analyses with and without this trial (Additional File 1 and Additional File 2). The only difference was that the cumulative meta-analysis became stable earlier (after inclusion of Gordon-Community) and then unstable after inclusion of Dalal (Additional File 1 - stability indicator = 0.0063 just greater than 0.005). All trials except Dalal contributed to the narrowing of the confidence interval over time centering on zero. Both of these are perhaps unsurprising given Dalal is the trial included with the most extreme result (except for DeBusk-Extended whose result is slightly more extreme but less precise).

## Discussion

### What does this study show?

This is the first attempt to apply these methods (cumulative meta-analysis and indicators of sufficiency and stability) to trials of cardiac rehabilitation. The trials included in this meta-analysis all contributed to reducing the difference and uncertainty in exercise capacity of home versus centre based rehabilitation. Kassaian and Dalal were the most influential because they had the most extreme results. The standardised mean difference continued to favour centre over home based rehabilitation but the size of that difference has narrowed over time, from 0.27 to 0.11. The confidence interval remained wide enough to include small to moderate differences [24].

The decision by the NIHR HTA programme to commission the BRUM trial in 2000 appears justified given that evidence available on the relative benefits of centre versus home based cardiac rehabilitation at the time was neither stable nor sufficient and included the possibility of large effect sizes (+/- 0.8 Cohen's criteria [24] - figure 2 cumulative SMD in 2000 -0.27, 95% CI -0.81,0.26). Of the six randomised controlled trials involving 517 patients that were available at this time (figure 1 studies before 2001) [14,17,18,20,22] only one of these had been conducted in the UK [14]. In 2007 just before BRUM published a further six similar trials had reported from six different countries [13,15,16,19,21,23]: USA, Canada, Italy, Turkey, UK and China. The HTA programme could not have known about these trials since trial registration of clinical trials only commenced on a voluntary basis in 2004 [25]. However even if these trials had been known of, it is not clear that they could have substituted for BRUM due to differences such as the form of home based rehabilitation [7], and trial size and duration.

### Comparisons with other studies

The indicators of sufficiency and stability presented here have been previously applied to five other case studies [5,26]. Two of these found the results were sufficient and stable. In the other three, the results were similar to ours - stable but not sufficient, with the estimate centering around the null effect and accumulating evidence simply narrowing the confidence interval around that null effect. In these three cases the authors concluded that carrying out further research in the area would be paramount to *"flogging a dead horse"*, with further studies unlikely to change the aggregate picture of a small effect.

### Limitations

The methodological focus of this paper had to do with assessing sufficiency and stability indicators using a case study in cardiac rehabilitation. The case study had two main limitations. The first was the heterogeneity of the types of home and centre based rehabilitation included within the trials. However, these trials were combined in a meta-analysis of exercise capacity in exactly the same way in the Cochrane systematic review and a meta-analysis confined to the three UK trials which used the heart manual [27] for home based rehabilitation [9,14,15] found three was too few for the sufficiency and stability analysis. The second limitation was the focus on a single outcome, exercise capacity, the only outcome common to all trials identified in the Cochrane review. However, exercise capacity is arguable the most plausible and key outcome for rehabilitation trials. Mortality data was only available from four studies and is likely to be confounded by drug treatment/uptake.

The results from a single case study have limited generalisability. More research is needed to better understand these indicators and the usefulness of the sufficiency indicator when applied to superiority comparisons showing differences close to zero. More case studies, simulations and Bayesian methods may be useful for this.

### Unanswered questions and future research

As all the trials included in this analysis were designed as superiority trials, we cannot conclude home based and centre based cardiac rehabilitation are equivalent. However, the above analyses show the difference in effect were relatively small and stable. Other factors have been shown to be important such as patient preferences [15]. The key question is what effect patients would consider worthwhile. Is the standardised mean difference of 0.11 sufficient for patients to choose one form of rehabilitation or the other. Only a study of patient preferences could answer this question.

## Conclusions

The methods used here seem promising and have implications for researchers, treating clinicians, payers, funders, sponsors, editors, ethics boards, patients, and the public. Sufficiency and stability measures can be calculated simply and shown graphically on a cumulative meta-analysis figure. They provide useful tools for considering whether further research is needed and the impact individual trials had on the meta-analysis. They are relatively straight-forward to calculate but not yet widely used. As with all meta-analyses, only published studies available at the time searches are conducted can be included. A policy-maker/funder wanting to use these methods to make funding decisions/assess research priorities would need to identify and consider ongoing studies. The thresholds suggested by Muellerleile et al are arbitrary and require further testing. In particular, rather than defining sufficiency mathematically with the focus on statistical significance, the benchmark should be based on substantive significance [28] set by patients' preferences. More case studies and further work to develop the sufficiency indicator would be helpful.

## Abbreviations

BRUM: The Birmingham Rehabilitation Uptake Maximisation Study (reference 9); CR: Cardiac rehabilitation; FDA: The United States Food and Drug Administration (http://www.FDA.gov); MEDLINE: MEDLINE is the National Library of Medicine's premier bibliographic database covering the fields of medicine, nursing, dentistry, veterinary medicine, the health care system, and the preclinical sciences; MET: Metabolic equivalent of task; NETSCC: NIHR Evaluation Trials and Studies Coordinating Centre (http://www.netscc.ac.uk); NICE: National Institute for Health and Clinical Excellence (http://www.nice.org.uk); NIHR: NHS National Institute for Health Research (http://www.nihr.ac.uk); NIHR HTA: NHS National Institute for Health Research Health Technology Assessment programme (http://www.hta.ac.uk); RCT: Randomised controlled trial; SD: Standard deviation; SE: Standard error; SMD: Standardised mean difference; UK: United Kingdom; USA: United States of America

## Appendix 1 - Calculation of the failsafe ratio for assessment of sufficiency

The failsafe ratio is calculated as the sum of the Z values from individual study results, compared to the number of studies with null results that would be required to make the meta-analytic result non-significant. It was derived by Muellerleile and Mullen based on Rosenthal's file drawer analysis [5,29]. It provides information about the amount of evidence against the null hypothesis and whether this weight of evidence is sufficient and unlikely to be changed with additional research. It is calculated as follows:

Where K_i _= the number of studies included in the meta-analysis at wave i

## Competing interests

JR and LD at the time of writing this paper were both employed by the NIHR Evaluation, Trials and Studies coordinating centre (NETSCC, http://www.netscc.ac.uk), who manage the NIHR HTA programme that funded the BRUM trial.

RT, KJ and JR were co-applicants for the BRUM trial.

## Authors' contributions

JR conceived the idea for this paper, RT and KJ provided advice and access to the data from the Cochrane review before publication. LD suggested the application of the sufficiency and stability indicators, carried out the statistical analysis and wrote the first draft of the paper. LD and JR re-drafted the paper and all authors critically agreed the final draft.

## Supplementary Material

Additional file 1 **Sensitivity analysis - Cumulative meta-analysis of studies excluding Kassaian**. This file shows the cumulative meta-analysis of studies published up to Jan 2008 comparing centre based versus home based cardiac rehabilitation using a random effects model and excluding Kassaian. The number of patients in this figure equals the cumulative number of patients included in each meta-analysis Click here for file

Additional file 2 **Sensitivity analysis excluding Kassaian - Cumulative meta-analysis from Additional File **1**with indicators of sufficiency and stability**. This file shows the cumulative meta-analysis from Additional File 1 with indicators of sufficiency (failsafe ratio) and stability (cumulative slope). 1. SMD = standardised mean difference (hedges g) 2. The cumulative SMD and 95% CI shown in this figure are exactly the same as those shown in Additional File 1, 3. Threshold for sufficiency > 1 (shown by red dashed line), not achieved in this cumulative meta-analysis 4. Criteria for stability < 0.005, achieved in this cumulative meta-analysis after inclusion of Arthur Click here for file
