# Supplementary figures and images for: "Flogging dead horses": evaluating when have clinical trials achieved sufficiency and stability? A case study in cardiac rehabilitation
Source: Trials. 2011 Mar 21;12:83. doi: 10.1186/1745-6215-12-83 (PMC3073877; doi:10.1186/1745-6215-12-83)

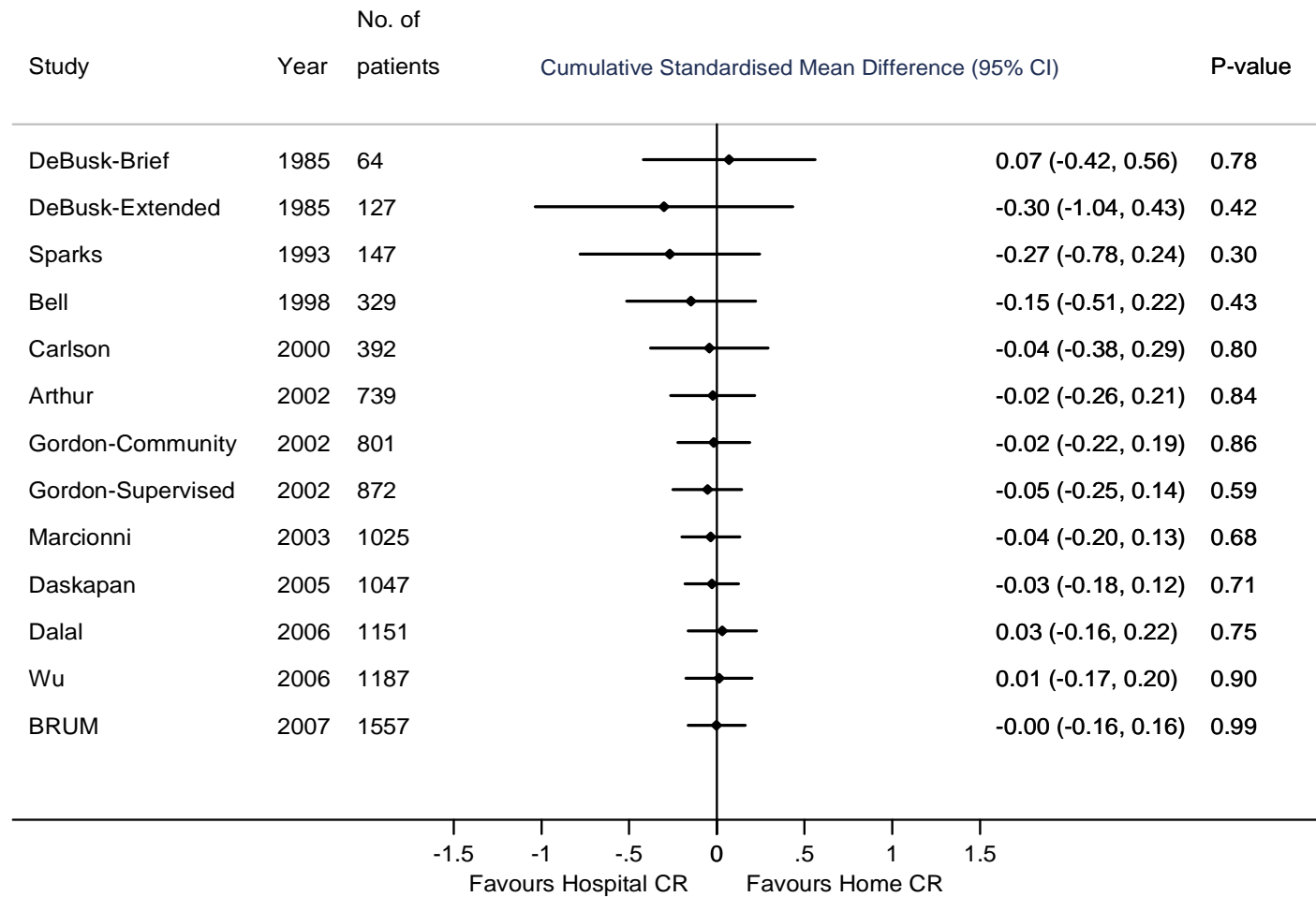

Supplement: Additional file 1 — Sensitivity analysis - Cumulative meta-analysis of studies excluding Kassaian. This file shows the cumulative meta-analysis of studies published up to Jan 2008 comparing centre based versus home based cardiac rehabilitation using a random effects model and excluding Kassaian. The number of patients in this figure equals the cumulative number of patients included in each meta-analysis [file 1745-6215-12-83-S1.PDF]
