# Supplementary material for: "Flogging dead horses": evaluating when have clinical trials achieved sufficiency and stability? A case study in cardiac rehabilitation
Source: Trials. 2011 Mar 21;12:83. doi: 10.1186/1745-6215-12-83 (PMC3073877; doi:10.1186/1745-6215-12-83)
Supplement: Additional file 2 — Sensitivity analysis excluding Kassaian - Cumulative meta-analysis from Additional File 1with indicators of sufficiency and stability. This file shows the cumulative meta-analysis from Additional File 1 with indicators of sufficiency (failsafe ratio) and stability (cumulative slope). 1. SMD = standardised mean difference (hedges g) 2. The cumulative SMD and 95% CI shown in this figure are exactly the same as those shown in Additional File 1, 3. Threshold for sufficiency > 1 (shown by red dashed line), not achieved in this cumulative meta-analysis 4. Criteria for stability < 0.005, achieved in this cumulative meta-analysis after inclusion of Arthur [file 1745-6215-12-83-S2.PDF]

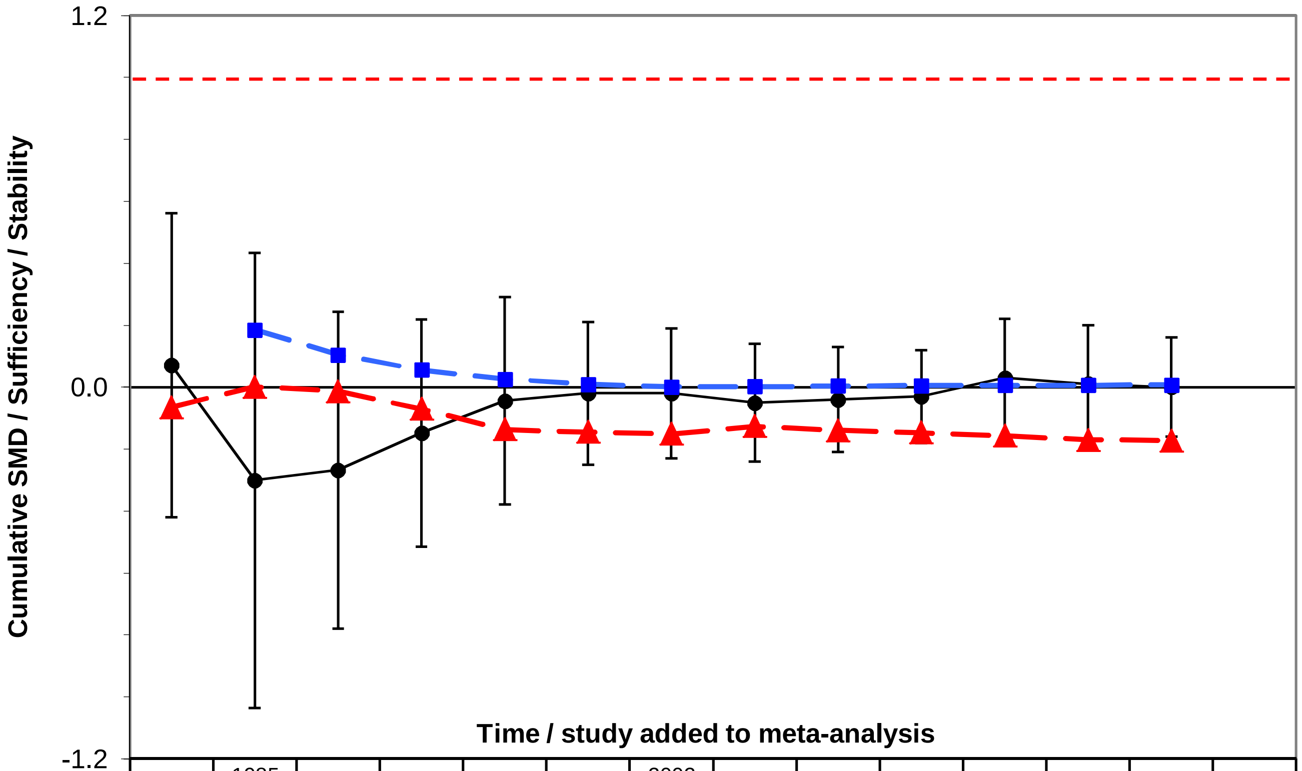

	1985 DeBusk-Brief	1985 DeBusk-Extended	1993 Sparks	1998 Bell	2000 Carlson	2002 Arthur	2002 Gordon-Comm	2002 Gordon-Sup	2003 Marcionni	2005 Daskapan	2006 Dalal	2006 Wu	2007 BRUM	
Cumulative SMD & 95% CI	0.07	-0.30	-0.27	-0.15	-0.04	-0.02	-0.02	-0.05	-0.04	-0.03	0.03	0.01	0.00	
Sufficiency	-0.065	0.001	-0.013	-0.071	-0.136	-0.145	-0.151	-0.126	-0.140	-0.147	-0.157	-0.170	-0.172	
Stability		0.185	0.105	0.056	0.026	0.0098	0.0014	0.0025	0.0045	0.0054	0.0063	0.0067	0.0069	
